# RECIST 1.1 assessments variability: a systematic pictorial review of blinded double reads

**DOI:** 10.1186/s13244-024-01774-w

**Published:** 2024-08-07

**Authors:** Antoine Iannessi, Hubert Beaumont, Christine Ojango, Anne-Sophie Bertrand, Yan Liu

**Affiliations:** 1 Cancer Center Antoine Lacassagne 33 Av. de Valombrose, 06100 Nice, France; 2 Median Technologies SA 1800 Route des Crêtes, 06560 Valbonne, France; 3 Imaging Center Beaulieu-sur-mer 18 Bd Eugène Gauthier, 06310 Beaulieu-sur-Mer, France

**Keywords:** Diagnostic errors, Statistics & numerical data, Quality improvement, Oncology, RECIST 1.1

## Abstract

**Abstract:**

Reader variability is intrinsic to radiologic oncology assessments, necessitating measures to enhance consistency and accuracy. RECIST 1.1 criteria play a crucial role in mitigating this variability by standardizing evaluations, aiming to establish an accepted “truth” confirmed by histology or patient survival. Clinical trials utilize Blind Independent Centralized Review (BICR) techniques to manage variability, employing double reads and adjudicators to address inter-observer discordance effectively.

It is essential to dissect the root causes of variability in response assessments, with a specific focus on the factors influencing RECIST evaluations. We propose proactive measures for radiologists to address variability sources such as radiologist expertise, image quality, and accessibility of contextual information, which significantly impact interpretation and assessment precision. Adherence to standardization and RECIST guidelines is pivotal in diminishing variability and ensuring uniform results across studies.

Variability factors, including lesion selection, new lesion appearance, and confirmation bias, can have profound implications on assessment accuracy and interpretation, underscoring the importance of identifying and addressing these factors. Delving into the causes of variability aids in enhancing the accuracy and consistency of response assessments in oncology, underscoring the role of standardized evaluation protocols and mitigating risk factors that contribute to variability. Access to contextual information is crucial.

**Critical relevance statement:**

By understanding the causes of diagnosis variability, we can enhance the accuracy and consistency of response assessments in oncology, ultimately improving patient care and clinical outcomes.

**Key Points:**

- Baseline lesion selection and detection of new lesions play a major role in the occurrence of discordance.
- Image interpretation is influenced by contextual information, the lack of which can lead to diagnostic uncertainty.
- Radiologists must be trained in RECIST criteria to reduce errors and variability.

**Graphical Abstract:**

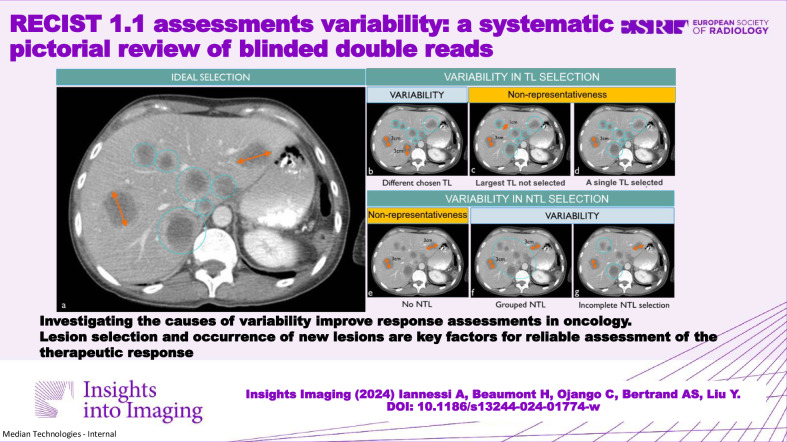

## Introduction

In the field of radiology, the absence of an indisputable “truth” in images can lead to different diagnoses based on the same image, resulting in inter-observer discordance of opinions.

In oncology, imaging-based evaluations are used to predict an accepted “truth” which is generally confirmed by histology or patient survival. Since this “truth” is not available at the time of analysis, errors can only be determined retrospectively. However, oncology evaluations are standardized using quantified criteria designed to decrease observers’ variability. Non-compliance with standardized reading instructions can be considered as avoidable errors, not just differences in opinions.

In clinical trials of cancer treatment efficacy, Blind Independent Centralized Review (BICR) with double reads is recommended by the Food and Drug Administration to control variability and avoid presumed investigator bias when the subject’s treatment assignment is known [1]. When discrepancies are detected between those radiologists, a third reader, known as an Adjudicator, is typically consulted to resolve the case and decide which of the two readers made the most accurate interpretation [2]. This reading paradigm also provides an opportunity to study the causes of inter-observer discordance during patient follow-up [3, 4].

In this analysis, we aim to systematize the causes of inter-observer variability in response assessments. We first describe the general construction of a quantified tumor burden follow-up criterion to identify the sources of variability in response assessments. Then, we focus on the four most significant variability factors identified during Response Evaluation Criteria in Solid Tumor (RECIST 1.1) evaluations in several centralized reviews [5]. By investigating the causes of variability, we aim to improve the accuracy and consistency of response assessments in oncology.

## Response evaluation criteria and RECIST 1.1 variability factors

In oncology, response evaluation criteria supply standardized reading rules to capture the evolution of cancer disease. These criteria have been shown to correlate with the gold standard of cancer treatment efficacy, which is ultimately “overall survival” [6]. Because Overall Survival is often a costly gold standard to refer to, in terms of patient benefits and trial resources, surrogate endpoints are accepted by drug approval agencies as primary endpoints in clinical trials [1]. The aim of Phase 2 studies is to evaluate treatment efficacy through a response objective, i.e., the “overall response rate”, hypothesizing that a decrease in tumor size theoretically correlated with a benefit on survival. Phase 3 trials confirm the benefit of a large population through a progression-free survival objective. A response evaluation criterion is designed to support those clinical trial aims. Commonly, response criteria define an initial baseline status which is then compared at fixed intervals over time [7]. The definition of the reference disease is quantified for one part and qualified for the other part. The semi-quantitative nature of baseline assessment lies in the fact that part of the disease may not be reliably measured, or measurement is too demanding. During follow-up, evaluations must be consistent with the baseline-defined disease and additionally, the appearance of new tumor lesions is scrutinized.

Considering how RECIST has been designed around three lesion categories, i.e., quantified target lesions (TLs), qualified non-target lesions (NTLs), and new lesions (NL), it is possible to break down its general causes of discordance related to the dimension of quantification and qualification [7].

Target lesions are all measured and their measured summed. The change of their measured sum is compared to the corresponding baseline and depending on a threshold of change, patients are diagnosed as stable, partially or completely responding, or progressing. The variability in TL selection at baseline or their measurements all along the process impacts the patient evaluation into the categories. Furthermore, the near-complete disappearance of lesions is subject to qualitative variability in evaluating the persistence of residual tumors or tissue considered scarred.

Non-target lesions are evaluated qualitatively. As for TL, their initial selection will impact the subsequent response and the evaluation of their complete disappearance is subject to divergent opinions, when display may correspond to scar tissue. In addition, lymph node sites that never disappear must be subjectively qualified as “non-pathological” to allow for a complete response. Regarding progression, in the absence of a formal quantified threshold, the subjective nature of the evaluation also leads to greater variability in the “significance” of progression.

New lesions are prone to observers’ variability in terms of detection and characterization due to their occasionally equivocal display.

Even if the oncological evaluation criteria are said to “quantify” the response, they remain subjective from several standpoints that impact the three main categories determining the overall response.

To summarize, we can consider three main criteria-derived factors of discordance between two radiologists which are: The initial lesions selection, the measurement of TLs, the qualitative evaluation of NTLs, and the detection of NLs (Table 1) [5].

**Table 1:** Categorical variability factors for “quantified” tumor assessment criteria

Lesion	Assessment (PD, PR, CR)	Variability factors
Target lesions	SOD↗ ≥ 20%/ SOD↘ ≥ 30%/ SOD = 0	• *Initial selection: number and location and organ belonging* • Measurement • *Qualitative assessment of the "disappearance" of small lesions*
Non-target lesions	Significant increase/completely disappeared	• *Initial selection: number and location and organ belonging* • *Qualitative assessment of the "significance" of the progression* • *Qualitative assessment of "non-pathological" status of nodes* • *Qualitative assessment of "disappearance" of small lesions*
New lesions	Absence/presence	• Detection (False Positive/False Negative) • *Qualitative assessment of "indisputability" (characterization)*

*SOD* sum of diameters, *PD* progressive disease, *PR* partial response, *CR* complete response. In italics, the subjective items of evaluation mostly related to a “characterization” task affect each lesion category and type of outcome

## RECIST variability in initial disease evaluation

The literature suggests that a large contribution to the response discrepancies is due to a difference in the definition of the disease at the baseline [8–10].

We illustrate hereafter the 3 causes of this baseline-derived variability.

### Ill-defined tumor selection criteria (Fig. 1)

Whereas RECIST gives clear selection rules for analyzing the disease at baseline, the cancer presentation however may be multi-metastatic so the choice of TL/NTLs, number, and distribution may vary between radiologists without infringing these rules [11]. Consequently, the “insufficient” whole disease representativity of the selection leads to response variability [12, 13].

**Fig. 1:**
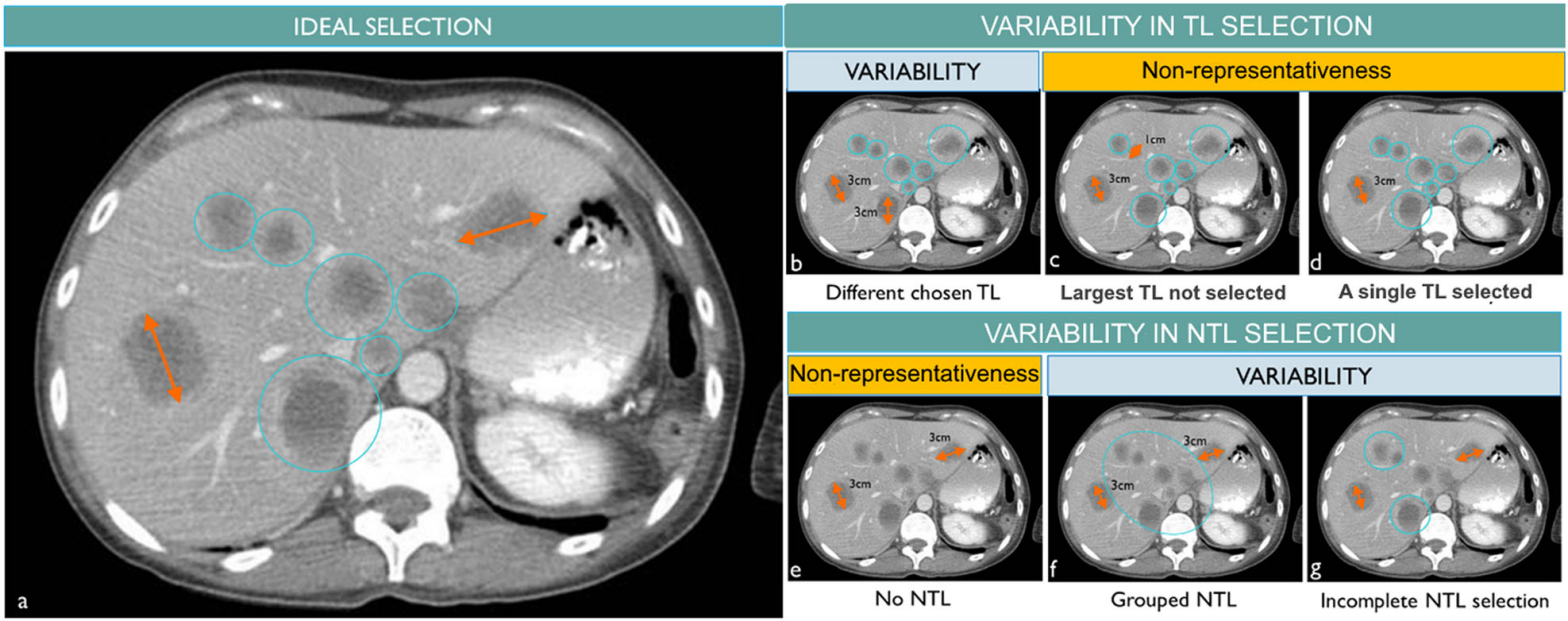
Acceptable reader variability of baseline selection considering RECIST 1.1 guidelines. The purpose of the baseline selection guideline is to standardize a fashion to best represent the disease by selecting the maximum and largest lesions (**a**). However, for practical use, the RECIST 1.1 criteria include room for variability in baseline assessment by limiting the number of target lesions (in red) to 2 per organ and 5 in total. Then, for patients with multiple lesions, there is a compliant inter-reader variability in the assessment (**b**, **f**, **g**). Besides the maximum number of lesions a justifiable reason such as equivocality of the finding, robustness of the measurement, or information about previously irradiated lesion must explain the variability but at the same time challenge the representativity goal of the selection task (**c**, **d**, **e**)

### Reader non-compliance with criteria (Fig. 2)

**Fig. 2:**
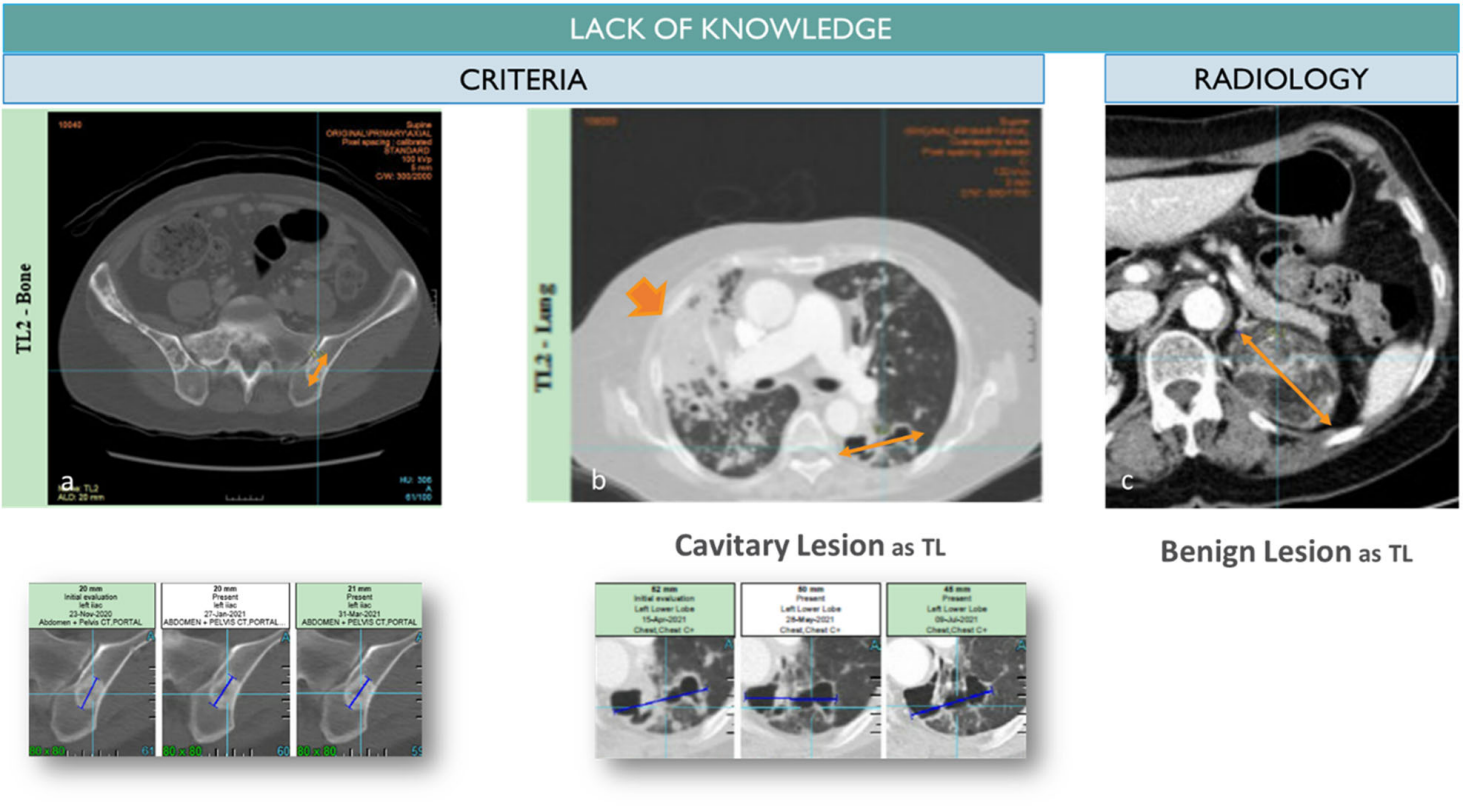
Baseline discrepancy caused by a lack of training. The lack of knowledge is not a frequent provider of variability between readers however often it results in errors of guidance when the reader does not master the criteria and selects bone blastic lesions as measurable lesions (**a**) or cavitary lesions while some more robust lesions for measurement is available (**b**). Rarely, the selection wrongly includes an unequivocally benign lesion due to a radiologist’s lack of knowledge such as the adrenal myelolipoma containing fat (**c**)

A non-compliance to the criteria’s rules of selection or measurability may be explained by a poor knowledge of the guidelines (Fig. 1), or occasionally by a flawed selection due to a lack of expertise in cancer imaging (Fig. 2) [9].

### Equivocal disease presentation (Fig. 3)

Due to healthy and diseased overlapped imaging presentation, sole imaging information can be insufficient to discriminate a malignant lesion from a benign finding leading to antagonist selections in trial with double reading. The selection of such equivocal findings by one of the readers as early as at baseline can lead to including in his tumor burden lesions that will remain stable over time and will impair the sensitivity of the response thresholds, Fig. 2 [14]. The impact of equivocal disease presentation is worse in the BICR setting with respect to routine-based RECIST evaluations where part of the patient’s clinical information is censored. Censored information that would have been helpful to clear a doubtful image such as historical data. Literature also demonstrates that the reader concordance of selection is lower for specific organ locations such as lymph nodes and peritoneal disease [15].

**Fig. 3:**
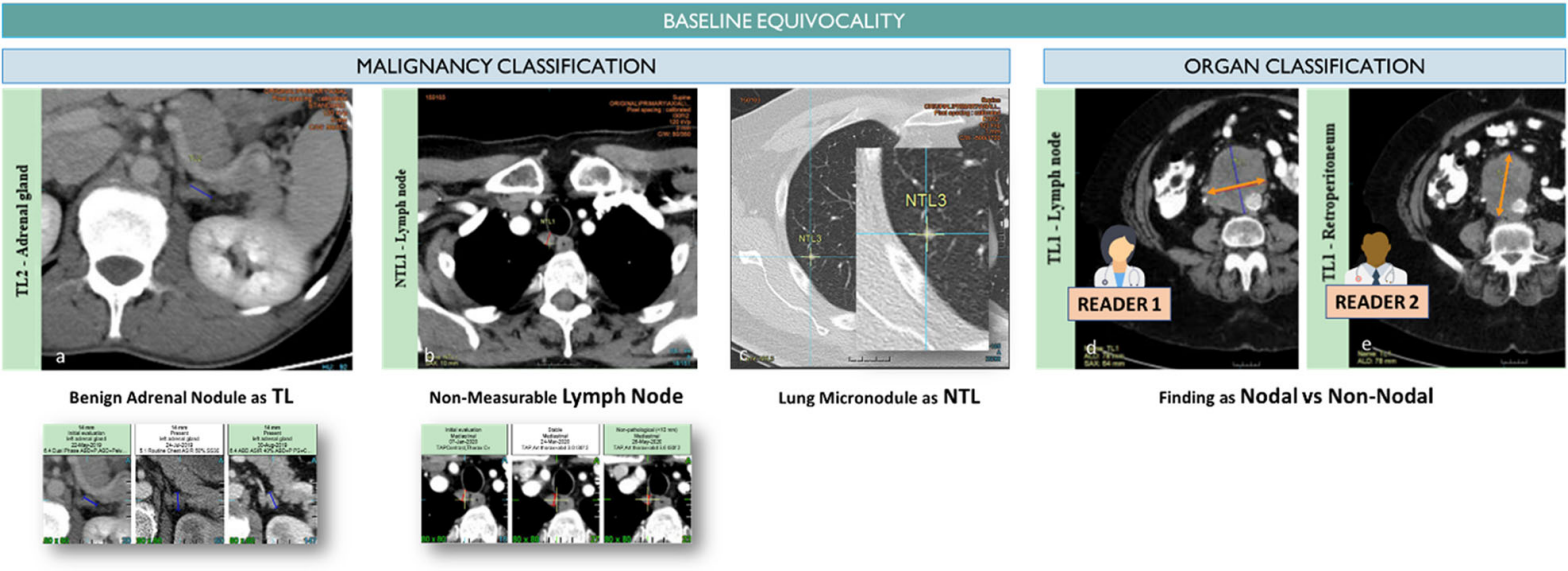
Baseline discrepancy caused by equivocality on the disease malignancy or the finding class. Equivocality at baseline is frequent due to the lack of previous examination or censored information for a blind evaluation. In such conditions, small lesions, e.g., adrenal nodules, juxta centimetric lymph node, and lung micronodules, might be wrongly considered as disease-related findings at baseline while they will remain stable over time and prove their benign characteristic (**a**, **b**, **c**). In some cases, the reader might select the same target but with a different organ classification (**d**, **e**). For RECIST 1.1, depending on how the same lesion is considered to be a lymph node or a mass, the specific method of measurement and threshold of the nodal lesion can impact the overall assessment

In addition to the uncertainty of malignancy, even for a lesion that is obviously cancerous, its belonging to a type of organ can be equivocal. It is frequently observed for intra-abdominal tissue nodules or neck nodules after surgery, which are reported variably as lymph nodes or carcinosis nodules. This classification affects the measurement method (short axis or long axis) and evaluation (pathological threshold for lymph nodes).

## RECIST variability in the recording of new lesions

The literature reports that 1/3 of discrepant detection of NLs during follow-up [16]. Four causes of discrepancy are illustrated below.

### False detections (Fig. 4)

The radiologist might wrongly consider a newly detected finding as an NL.

**Fig. 4:**
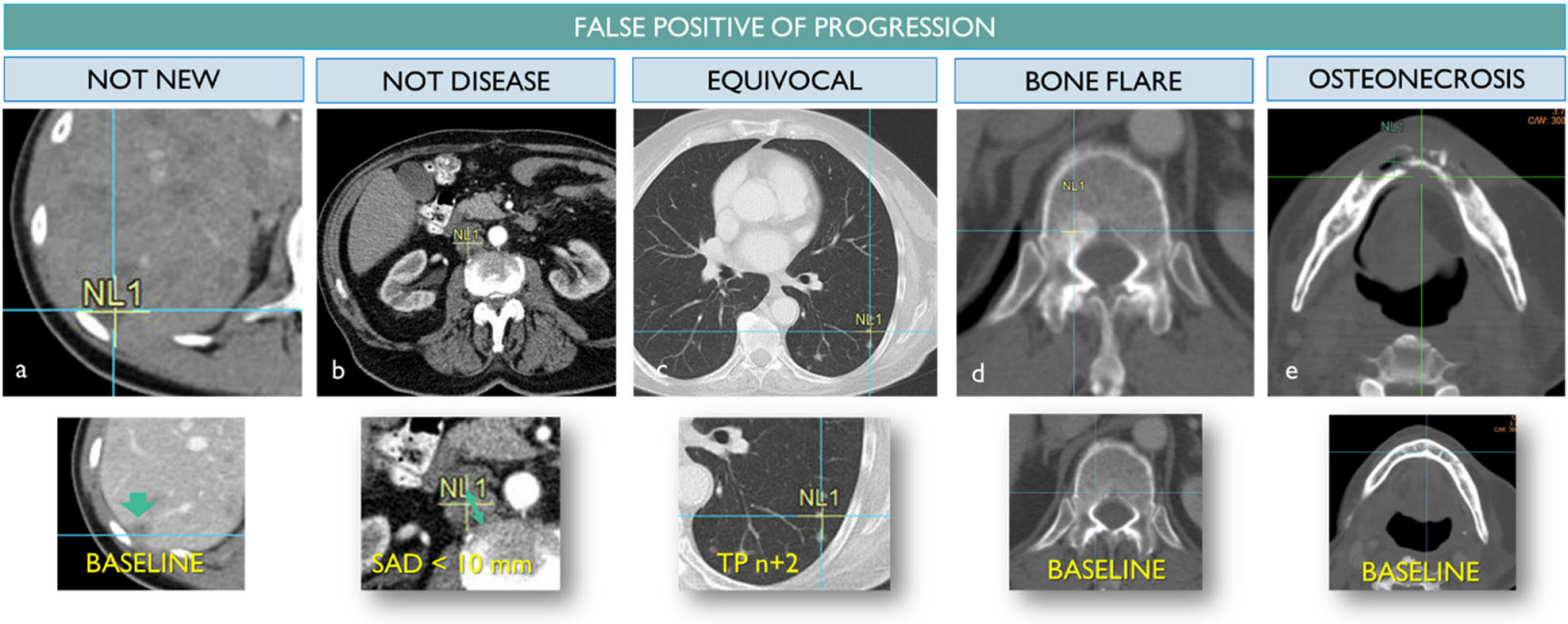
False alarm for progression. From left to right, to define a new lesion, the finding should be new and not present in the baseline (**a**), considered pathologic so ≥ 1 cm for lymph nodes (**b**), unequivocally related to the cancer disease spreading unlike a micronodule possibly linked to intercurrent inflammation (**c**), not related to a blastic bone healing phenomenon (**d**) or a treatment-induced adverse event such as osteonecrosis of the jaw (**e**)

To qualify for an NL, a finding must be unequivocally “new”, “present”, and “cancerous”.

Indeed, it should be noted that an NL can only be defined as such if it is not present at baseline because otherwise, it belongs either to the TL or to the NTL group and benefits from qualitative or quantitative follow-up.

Also, if an NL is small, it could be a result of an artifact or a higher image definition compared to the baseline.

Ultimately, a new and indisputable anomaly is not necessarily cancerous during RECIST follow-up:

- An intercurrent lung consolidation may mimic an NL.
- The malignant and reactive lymph nodes are a differential diagnosis that can lead to variability. Therefore, RECIST specifies a threshold of 10 mm short axis to be considered lymph node disease [17].
- Treatment-related changes must be known. The “bone flare” phenomenon has been documented for a long time and it leads to the condensation of sometimes subtle bone lesions initially mimicking the appearance of new condensing lesions [18]. Only the appearance of a lytic or extraosseous tissue lesion should be considered as an NL.

The reporting of an NL during follow-up leads to classifying the corresponding patient as progressing, triggering early discontinuation of his treatment. In this respect, the RECIST 1.1 criterion is conservative. In ambiguous situations, the guidelines propose to be decided through later follow-up. This follow-up allows us to confirm the progressive nature of the finding with more certitude.

### Non-detections (Fig. 5)

Non-detection during one or more time points is observed and involves usual sites of metastasis such as the liver or the lung. On the other hand, more often the discordant sites are either in the bone or in less frequent metastatic sites [19]. It is possible that a tunneling bias of attention plays an important role in these occurrences [20]. Indeed, bone lesions mostly are low conspicuity because they are lytic or sparsely dense in a dense environment, so they require an active search by reformatting and adapted windowing because otherwise, the radiologist cannot see them. Also, unexpected sites such as first slices or soft tissue are responsible for non-detection [21].

**Fig. 5:**
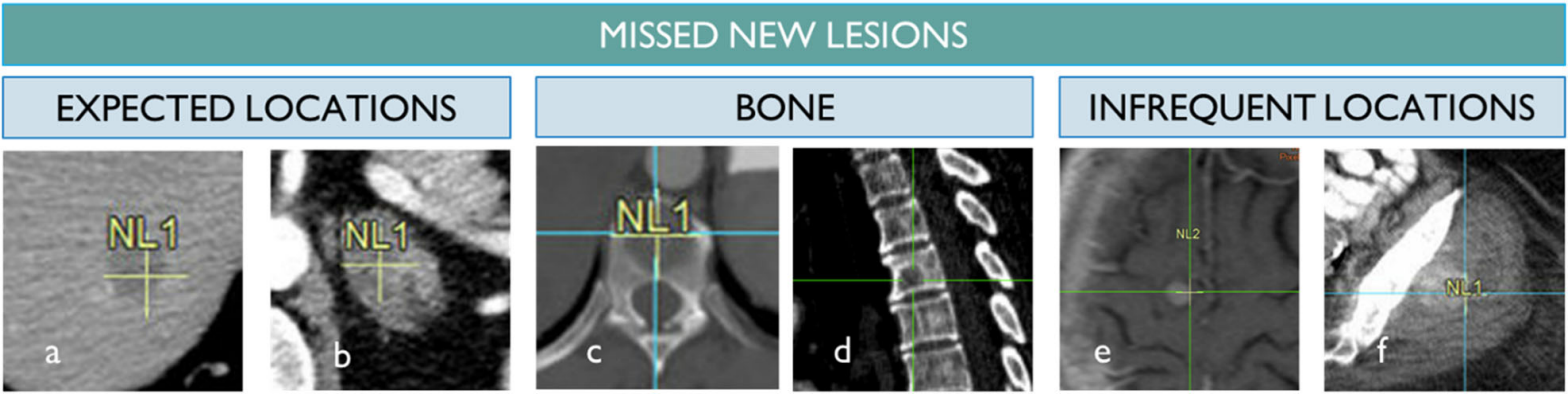
False Negative for Progression. The most frequent metastatic sites for lung cancer depend on the primary site, such as the liver and adrenal gland (**a**, **b**). While these lesions can be missed, more often, new lesions are missed when they are located in infrequent locations like the bone due to their low conspicuity in axial view (**c**) compared to sagittal reformats (**d**). Infrequent locations, such as the brain or soft tissue, are unexpected and more susceptible to attention bias (**e**, **f**)

### Under-detections

Under-detection corresponds to incomplete detection of several NLs. This phenomenon has theoretically little impact on a discordant response according to RECIST since a single NL is sufficient to trigger progression. This has been well documented and corresponds to 20% of evaluation errors in oncology. It reflects a premature loss of attention by the radiologist after the first phase of successful research and is frequently described as “satisfaction of search” bias [22].

### Delayed-detections (Fig. 6)

It is common to observe discordance in the date of progression between two readers, leading to different progression-free survival rates (around 30% in the literature) [19]. Part of the explanation lies in the size of the NL, which becomes more visible over time, reducing the risk of perception discordance. Another part of the explanation lies in the unacknowledged subjectivity of the RECIST criterion about the significance of disease progression. It is possible that the radiologist does not identify a new anomaly because, in their opinion, it does not represent sufficient progression based on the rest of the evolution and is therefore somewhat equivocal. Delayed detections frequently involve isolated appearances of equivocal findings, such as lung micronodules and centimeter-sized lymph nodes.

**Fig. 6:**
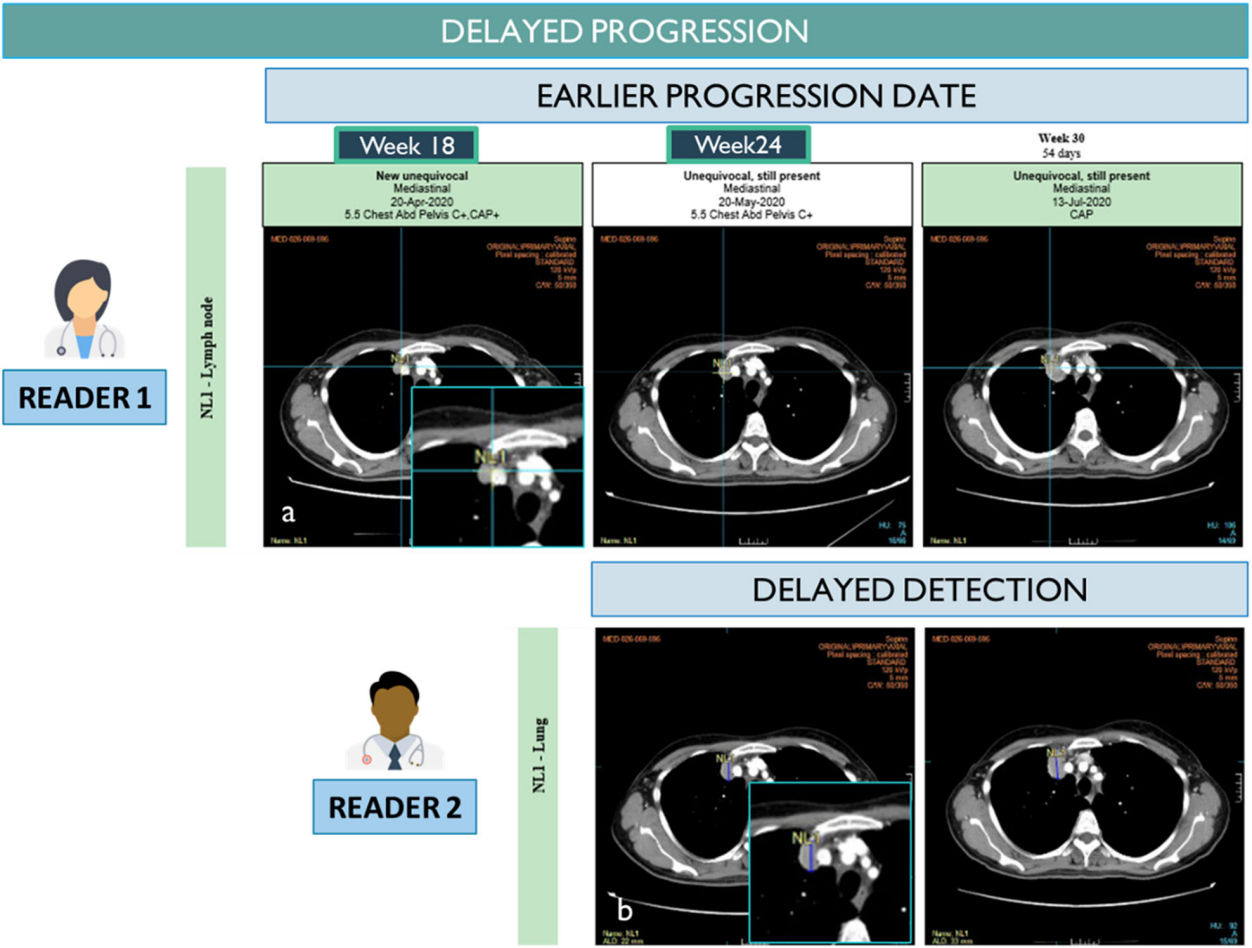
Delayed detection. The two radiologists identified the same new lesion (**a**, **b**) but radiologist 2 detected it one visit later leading to a 6 weeks discrepant date of progression

## RECIST variability in measurements of the lesion (Figs. 8–11)

The baseline introduces a measurability cutoff of 1 cm for solid tumors to qualify as TL versus NTLs. This rule is supported by a reduction in reader variability and the expected shrinkage of tumors after treatment, requiring a minimal initial tumor burden to interpret changes versus variability [23]. It should be noted that the “measurability” criterion applies per lesion, but no measurable criteria apply to the patient. For this reason, patients are eligible for quantification from at least one lesion of 1 cm, which raises questions about the variability of response assessment in patients with smaller tumor burdens compared to those with larger tumor burdens.

**Fig. 7:**
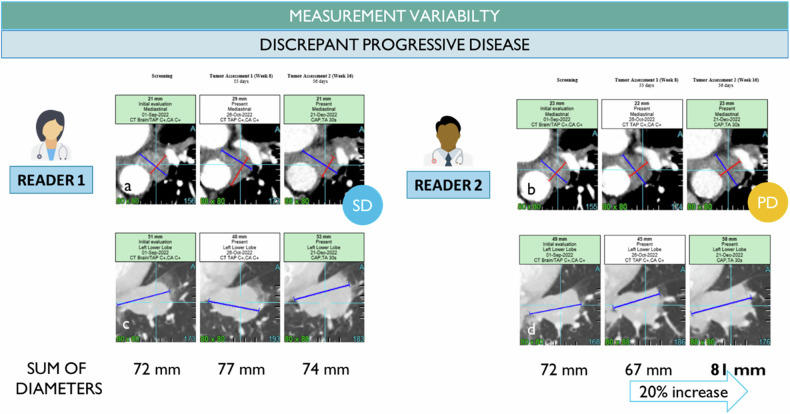
Inter-observer measurement variability. At baseline, the two radiologists selected the same targets as mediastinal node (**a**, **b**) and lung nodule (**c**, **d**) with variable measurement at the lesion level but compensated when summing the index lesions (identical sum of diameters). Then, during the follow-up, the measurement variability leads to a patient response discrepancy with progressive disease (20% increase) assessed by reader 2 versus non-progressive disease for reader 1 while the difference of the sum of diameter at the visit 1 is 10 mm and at visit 2 is 7 mm

During follow-up, the same use of threshold explains that even a millimetric difference between two radiologists can be responsible for a discrepancy between progression and stable disease, and even progression and complete response, especially after a response when tumor burden includes lymph node elements (Fig. 7).

**Fig. 8:**
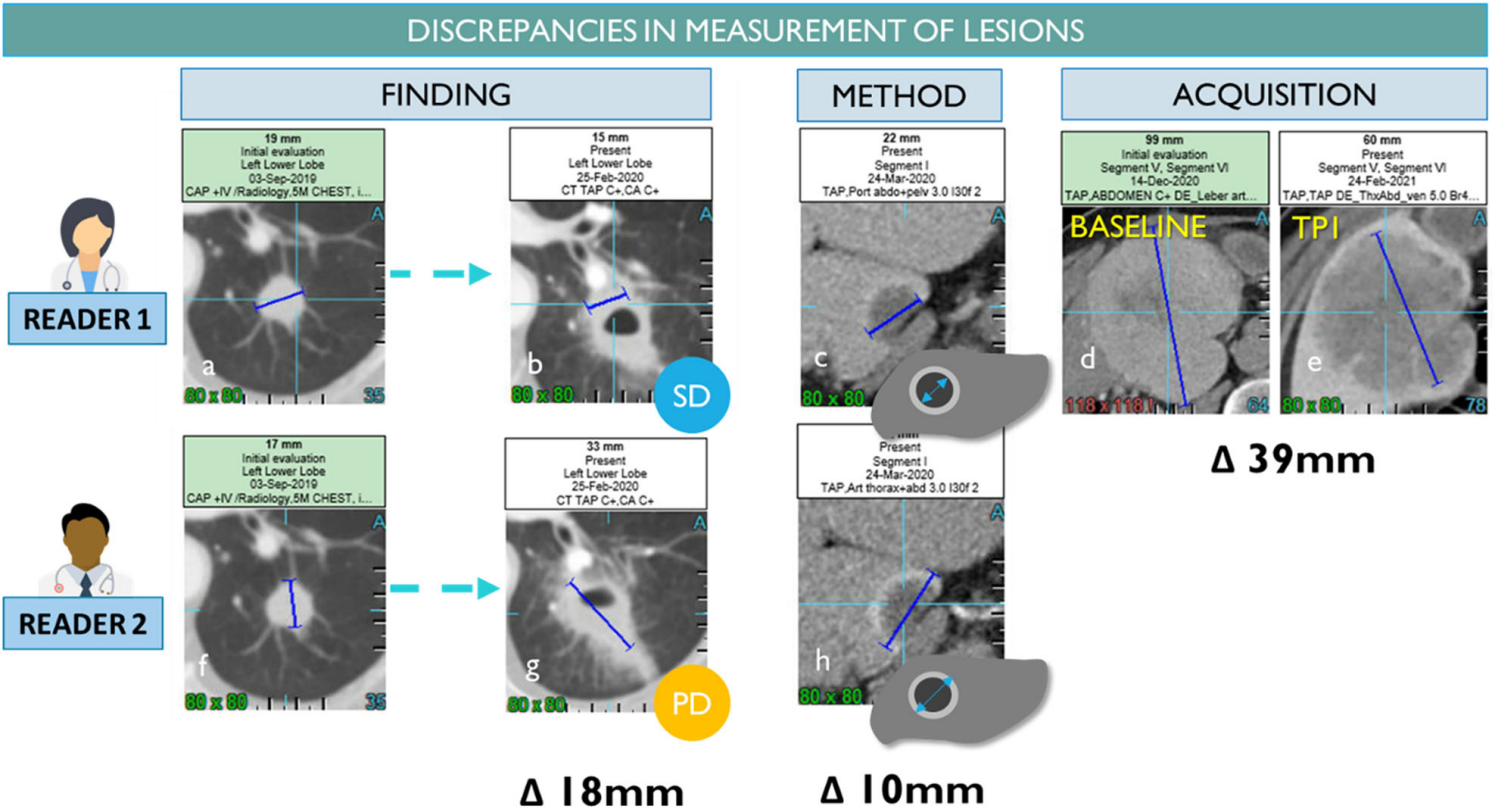
Measurement variability factors (inter and intra-observers). The discrepancy in measurements can lie inside the finding such as cavitation or speculation and difficulty of measurement (**a**, **b**, **f**, **g**), the method of measurement such as rim included or not in the measurement (**c**, **h**), the selection of a different phase of injection even for a same reader during follow-up (**d**, **e**)

Measurement is repeated for each timepoint and the sources of the sum of diameter variability are illustrated in Fig. 8 and can be broken down as follows [24]:

- Disease distribution, location, finding appearance (e.g., single or multiple lesions, type of organ such as head and neck are difficult to delimitate with respect to lung nodules) [25, 26]
- Non-standardized measurement method (e.g., measurement rules for cavitary lesions)
- Inappropriate windowing
- Inherent reader variability (i.e., inter-observer variability higher than intra-observer) [15]
- Inconsistent choice of series for annotations (e.g., inconsistent acquisition times) [16]
- Poor image quality (cf. discussion)

**Fig. 9:**
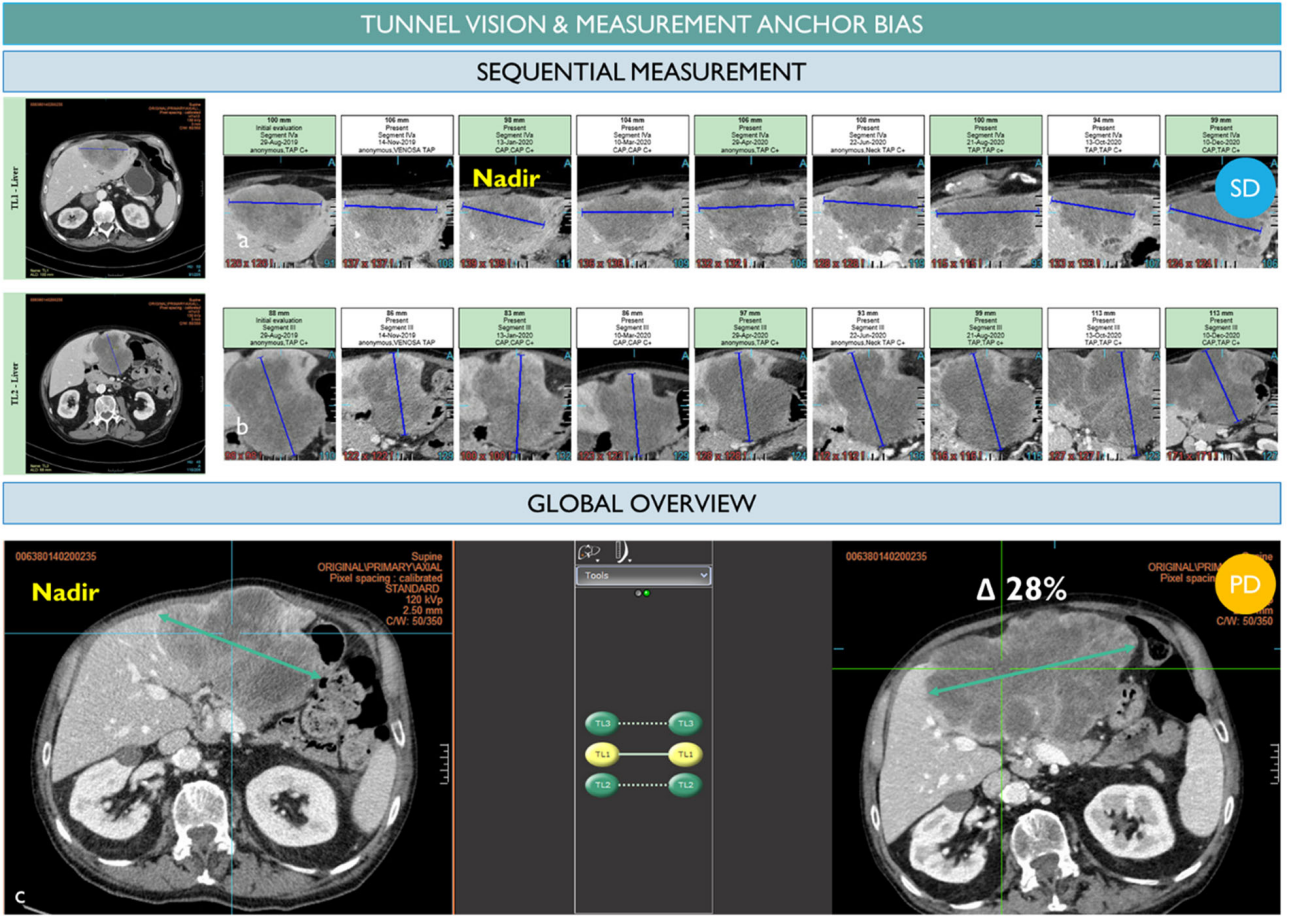
Anchor bias in measurement. The radiologist chose 2 targets in the liver and his assessment concluded to be a stable disease after 9 evaluations (**a**, **b**). However, with a retrospective look, one can visually observe a significant increase in the same target measured (**c**). We suspect a confirmation bias in the measurement workflow timepoint after timepoint

Furthermore, in RECIST analysis, those repeated measurements are typically performed sequentially in a workflow, and this can make radiologists vulnerable to confirmation bias, leading to a tendency to reconfirm previous measurements [27]. As a result, the overall change in lesion size may be minimized (as shown in Fig. 9). If a radiologist works differently and systematically with respect to the baseline or nadir, the measured lesion size may differ accordingly.

**Fig. 10:**
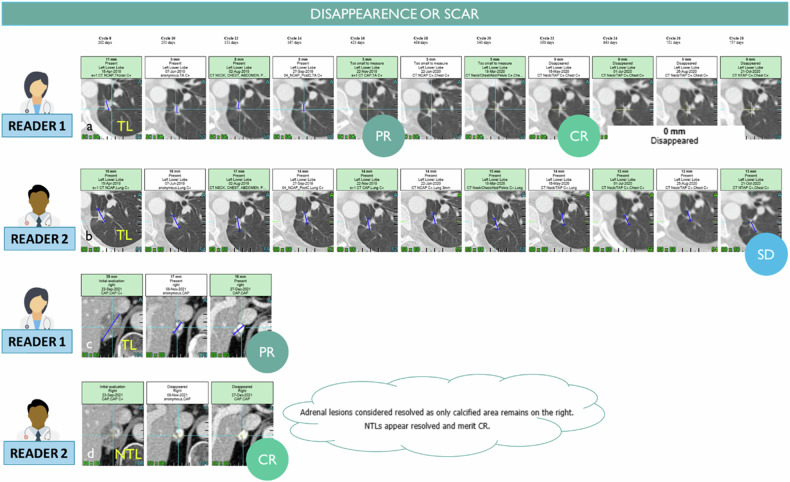
Discrepancy on remaining visible findings after treatment. The same lung nodule target was chosen by two radiologists (**a**, **b**). Radiologist 1 considers the remaining visible finding as a scar while radiologist 2 continues to measure the remaining disease (18 mm) preventing a complete response for this patient. The same adrenal gland finding was chosen as TL by reader 1 and NTL by reader 2 (**c**, **d**). Unlike reader 1, reader 2 considers that the lesion disappears with remaining calcified scars although it is still visible and measured by reader 2

Finally, the discordance of responses is seen when lesions have become very small without complete disappearance. The measurement of a small residual lesion for which the measurement can be continued indefinitely without obtaining a complete response. In this case, the lesions no longer decrease but no longer grow and could correspond to a scar (Fig. 10). In characterizing whether a complete response has been achieved, the radiologist’s judgment is therefore necessary to consider the overall level of response. In practice, the discrepancy between PR and CR is less important from a clinical trial perspective since the overall response rate is calculated on the whole PR + CR, and the date of the first response is not influenced.

## RECIST variability in the assessment of the not-measured disease (Fig. 11)

First, the evaluation of the NTL is subjective as it concerns the truly non-measurable diseases such as blastic bone metastasis and the not-measured baseline residual disease after TL selection. Also, NTLs are sometimes grouped together to ease the follow-up record of diffuse disease (Fig. 1).

**Fig. 11:**
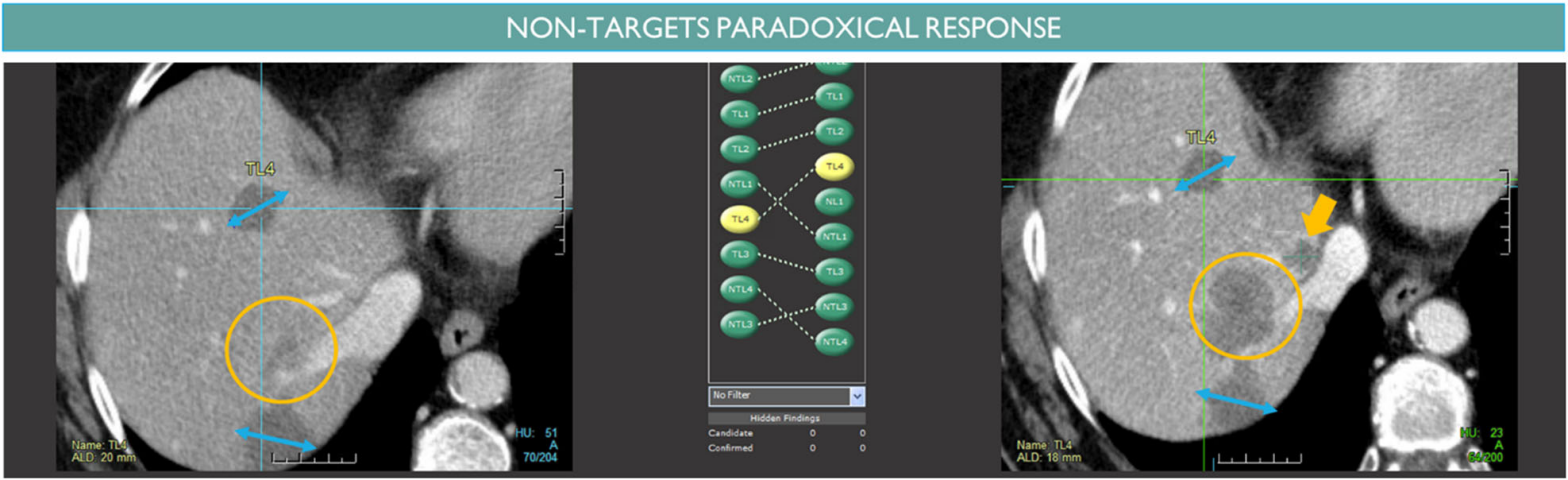
Paradoxical response of non-targeted disease. The measured disease is stable (double arrowhead) while the non-targeted disease (circle) increases unequivocally. A small new lesion can also be identified (arrow)

Dissociated response is defined as the coexistence of responding and non-responding lesions within the same patient. It is particularly observed with immunotherapy where such a paradoxical response between TL and NTL (Fig. 11) can be a difficulty [28, 29]. Unlike TL, the regular workflow that consists of comparing lesions to their Nadir is not well suited for monitoring NTLs as it is tricky to identify the Nadir of NTL without plotting a quantified measure of their changes. Therefore, in practice, NTL evaluations are compared to the last available examination.

Second, the evaluation of each NTL is aggregated subjectively into an NTL category response and RECIST states that NTL assessment is a function of the overall disease and therefore should consider the evolution of TLs and the presence of NLs [30].

Because of these above different factors, there is inter-observer variability in the evaluation of NTL categories responsible for discordance in the overall response. As explained, this occurs even more when the TL/NTL response is dissociated.

Previously mentioned difficulties in assessing complete response versus non-complete response for small remaining lesions versus scars also concern the NTL category (Fig. 11). However, even larger lesions are concerned for non-measurable bone lesions when a lytic lesion heals as a blastic lesion (bone flare) or blastic lesions persists throughout the evaluation with no increase (Figs. 2a and 5d). in such case, the assessment of complete response versus remaining bone metastasis is subjective. The same difficulty in distinguishing CR versus non-CR for cystic or cavitary lesions after treatments (Fig. 2b).

## Discussion

In our pictorial overview, we highlight that each response category in the RECIST criteria is subject to significant sources of reader variability. The expertise of the radiologist certainly plays an important part [31] such as the patient assessment performed by a single observer [32]. Therefore, for this observer, we propose to summarize individual preventive actions that radiologists may find valuable into a “toolbox,” as detailed in Table 2 [33].

**Table 2:** Radiologist RECIST 1.1 toolbox of preventive action

Selection of lesions	• Expertise and training on the criteria’s read rules for follow-up.
	• Comprehensiveness and Representativeness of the choice
	• Ensuring non-equivocality of lesions, particularly for target lesions
Detection of new lesions	• Always checking the baseline to confirm the absence of new lesions
	• Measuring juxta centimetric lymph nodes
	• Confirming equivocal and micro-lesions using follow-up imaging
	• Using a checklist to avoid in attentional bias and actively search in certain areas not expected
	• Including bones on the checklist:
	○ Beware of the appearance of new condensed bone lesions and recognize the bone flare-up effect
	○ Using sagittal and coronal reformatting with bone windowing
	• After detecting a new lesion:
	○ Always looking for a second metastasis finding the first one
	○ Checking previous examinations to precisely date the progression of the disease
Assessment of non-target lesions	• Performing a comparison of the current examination with the baseline and an intermediate timepoint to verify the absence of dissociated response to the targets and to get a more global view of the evolution.
Measurement of targets	• Measuring with the same method throughout the analysis
	• Using the same injection time for the measurements
	• Performing a comparison of the current examination to the baseline and an intermediate nadir to get a global perspective and avoid confirmation bias.
	• Utilizing semi-automatic measurement tools when available
	• Not measuring a target when the quality for robust measurement is not met (non-evaluable)

List of items to control variability and discordance rate

The literature provides figures on the causes of RECIST variability, which can help prioritize actions using a Pareto strategy based on the context of the evaluation [34, 35]. In phase 3 or adjuvant settings of clinical trials, progression is the key event of interest, and strategies to minimize variability may focus on assessing NLs. Conversely, in phase 2 trials, the primary event of interest is the first response, and thus, the measurement of the disease and choice of lesions are critical components of the assessment that require close monitoring.

In addition to the radiologist-related causes of variability, we should consider two other sources of variability related to the information provided to those observers.

**Fig. 12:**
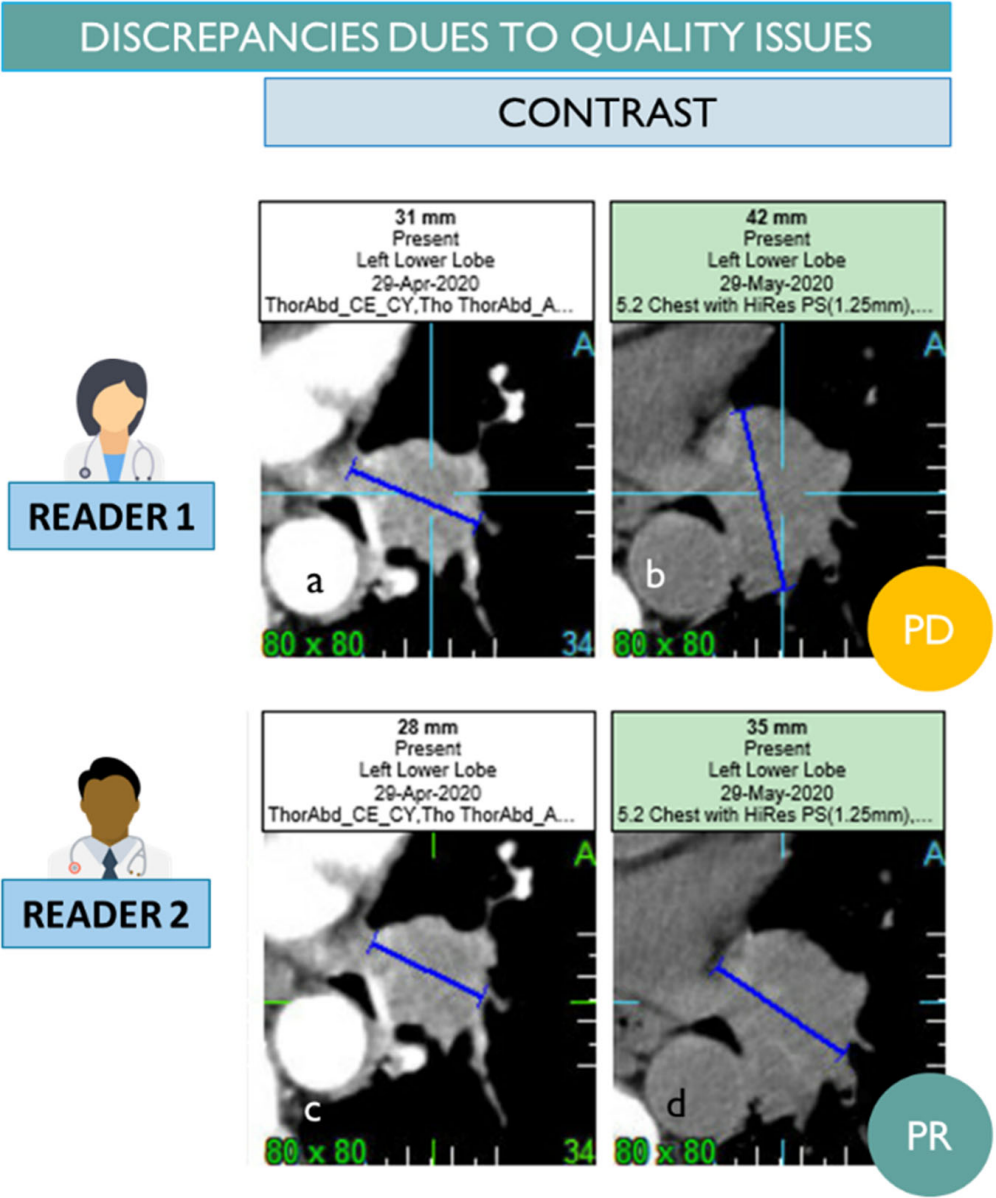
Image quality-related discrepancy. One lung para-mediastinal target is measured with mediastinal windowing and good visualization of adjacent vascular structures (**a**, **c**). During the follow-up, one CT evaluation is performed without contrast injection (intercurrent contra-indication). The higher inter-reader variability of measurement due to the lack of contrast in the image resulted in a progression discrepancy (**b**, **d**)

The first source is related to the quality of the images provided for assessment [36]. To comply with the RECIST 1.1 assessment, the images should meet specific minimal requirements for acquisition, such as slice thickness. Standardization is also necessary to ensure that images from the same patient are acquired under the same conditions. In a multi-site study, standardization between sites can be challenging, even though the evaluation using a chest abdomen pelvis used for size measurement is less of a problem than quantitative metabolic assessments. To address this issue, acquisition charters are provided to sites taking part in clinical trials to help standardize the acquisition process. The quality of the image can also be deteriorated by patient-specific factors such as contraindications that may prevent the injection of contrast agents or prosthetic implants that can cause image artifacts (Fig. 12), making a robust analysis difficult. Despite these challenges, it is the responsibility of the radiologist to provide an assessment based on the images provided, considering the level of trust they have in the images.

The second source of variability is related to contextual information that may or may not be accessible to the radiologist. During central analysis, radiologists are often blinded to clinical information and historical examinations, which can introduce uncertainty into radiologic findings and result in variability in the assessment. This lack of information can make the selection of liver findings at baseline challenging, as the radiologist may not have the knowledge of their evolutive character to relate them to metastasis in the absence of a typical benign appearance. In contrast, when assessing patients at the hospital, the radiologist may be influenced by contextual information, leading to an optimistic bias, particularly for response assessments [16, 37].

## Conclusion

This pictorial provides a comprehensive overview of the variability in RECIST assessments, with two significant impacts: it affects study endpoints during drug development and influences clinical decisions in patient management. Discrepancies in the detection of NLs can lead to inconsistent assessments of disease progression, potentially influencing the overall outcome of the study. Moreover, the accuracy of assessments relies heavily on adequate additional clinical information, emphasizing the importance of considering the reader variability when making clinical decisions based on RECIST assessments. Reader variability is inherent in radiologic oncology assessment. RECIST 1.1 criteria help to control this variability by standardizing evaluation rules to produce comparable results between different studies. Being compliant, the radiologist’s primary objective is to minimize the risk factors contributing to variability. During the process of double reading for central assessments, this variability is materialized into discordance rates which are informative of the origin of the reader’s variability. The baseline and target selection plays a major role in the occurrence of discordance. The appearance of NLs is also a determining factor in terms of both detection and characterization discrepancies. It seems important to always reconsider the presence of an equivocal lesion at baseline or during follow-up in order not to distort the calculation of the initial lesion sum or to declare a progression too early. The evaluation of serial radiological examinations two by two may favor the confirmation bias while intermediate and global retrospective reviews help to remove this bias for a better understanding of the overall patient response. The interpretation of images can be influenced by contextual information, and the lack of such information can lead to uncertainty in the assessment. Conversely, the presence of specific information can result in a biased assessment.

### Supplementary Information

ELECTRONIC SUPPLEMENTARY MATERIAL

## Data Availability

Not applicable.

## Abbreviations

BICR: Blinded Independent Central Review
NL: New lesion
NTL: Non-target lesion
RECIST: Response Evaluation Criteria in Solid Tumor
TL: Target lesion
