# Supplementary material for: RECIST 1.1 assessments variability: a systematic pictorial review of blinded double reads
Source: Insights Imaging. 2024 Aug 7;15:199. doi: 10.1186/s13244-024-01774-w (PMC11306910; doi:10.1186/s13244-024-01774-w)
Supplement: Supplementary file 1 — ELECTRONIC SUPPLEMENTARY MATERIAL [file 13244_2024_1774_MOESM1_ESM.pdf]

RECIST 1.1 assessments variability: a systematic pictorial review of blinded double reads

ELECTRONIC SUPPLEMENTARY MATERIAL

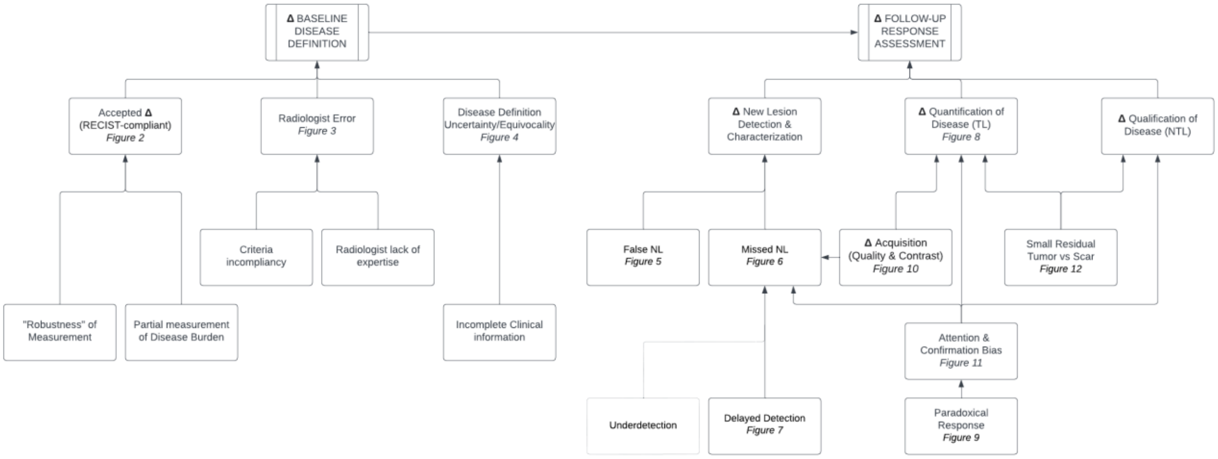

Annexed Figure: Typology and causation of variabilities of RECIST 1.1 assessments. Baseline variability (Δ) is a potential cause of response variability, which can be divided into accepted, non-accepted, and inevitable variability. The latter is often related to an equivocal presentation of the disease, which can be exacerbated by incomplete contextual information. Response assessment variability, on the other hand, is caused by variable assessment of new lesions (NL), target lesions (TL), and non-target lesions (NTL). Variability in acquisition and artifacts may increase the variability of NL or TL assessment, respectively, regarding their detection or measurement. Cognitive bias can also cause assessment errors, commonly due to confirmation and tunnel bias, which are particularly susceptible to dissociated responses between TL and NTL categories. Small residual TL or NTL versus complete disappearance is also a cause of variability for those concerned categories.
